# Pulmonary mucoepidermoid carcinoma with local intrabronchial invasion in a young lady: A case report of successful management via uniportal VATS sleeve lobectomy

**DOI:** 10.1016/j.ijscr.2025.111895

**Published:** 2025-09-06

**Authors:** Rawand Abdulrahman Essa, Diyar Saddam Sulaiman, Kalthuma Saleh HamadAmen, Saman Salaheldeen Abdulla, Rebaz Hamza Salih, Luqman Rahman Sulaiman

**Affiliations:** a University of Kurdistan Hawler, School of Medicine, Erbil, Kurdistan region, Iraq; b Rizgary Teaching Hospital, Erbil, Kurdistan region, Iraq; c Hawler Medical University- College of Dentistry, Erbil, Kurdistan region, Iraq; d PAR Hospital, Erbil, Kurdistan region, Iraq; e Hawler Medical University- College of Medicine, Erbil, Kurdistan region, Iraq

**Keywords:** Pulmonary Mucoepidermoid carcinoma, Intra-bronchial, Local invasion, U-VATS, Sleeve lobectomy

## Abstract

**Introduction and importance:**

Mucoepidermoid carcinoma (MEC) is an extremely rare type of lung cancer, accounting for approximately 0.2–0.4 % of all lung cancers. It typically presents as a central airway lesion, with symptoms such as cough, hemoptysis, bronchitis, wheezing, fever, chest pain, and clubbing of the fingers. Histologically similar to tumors in major salivary glands, it mostly arises from small salivary glands that line the tracheobronchial tree.

**Case presentation:**

We have reported a rare case of intrabronchial mucoepidermoid carcinoma that extended extra-bronchially in a 25-year-old female presenting with hemoptysis for 10 days associated with mild cough and wheezing. A computerized tomography (CT) scan revealed a 3.5 cm lesion in the right upper bronchus, and fiber optic bronchoscopy confirmed an intrabronchial lesion. We performed right sleeve lobectomy via a novel Uniportal video-assisted thoracoscopic surgery (U-VATS) approach.

**Discussion:**

Since Mucoepidermoid carcinoma (MEC) frequently manifests with vague symptoms, diagnosing it when it spreads from extra-bronchial intrabronchial can be difficult and uncommon. The typical histology of Mucoepidermoid carcinoma (MEC) composed of varying 3 cell types, but the histology of our case was single cell type and it was diagnosed by genetic analysis in another advanced hospital. The effectiveness and safety of Uniportal video-assisted thoracoscopic surgery (UVATS) have been acknowledged globally. Shorter surgical times, less pain, and a decreased requirement for extensive chest tube drainage are all linked to this technique.

**Conclusion:**

This case illustrates how a young woman's intrabronchial mucoepidermoid carcinoma was successfully treated with a sleeve lobectomy performed via Uniportal video-assisted thoracoscopic surgery (VATS).

## Highlights

• An Extremely rare case of intrabronchial mucoepidermoid carcinoma (MEC) extended extra-bronchially in a young lady.
• Novel surgical approach: which was right sleeve lobectomy via uniportal video-assisted thoracoscopic surgery (U-VATS).

## 1 Introduction

Mucoepidermoid carcinoma (MEC) is a rare form of lung cancer, making up only about 0.2–0.4 % of all lung cancers [1,7]. It usually appears as a lesion in the central airways, with symptoms that include cough, hemoptysis, bronchitis, wheezing, fever, chest pain, and finger clubbing [7]. While the tumor typically grows slowly, it can spread to lymph nodes or invade local tissues [4,8]. MEC is histologically similar to tumors found in major salivary glands and usually originates from small salivary glands that line the tracheobronchial tree [5,7,10]. The primary treatment for MEC is surgical resection, of which lobectomy is the most frequently performed procedure [2]. Our case presented with subacute hemoptysis with mild cough and wheezing which it has unique presentation and the tumor's characteristics may give valuable insights into the disease. Mucoepidermoid carcinoma (MEC) is more common in older adults, but we have found it in a young lady. MEC typically occurs in the salivary gland but in our case, it is a lung tumor. The noteworthy of this case is the combination of a rare tumor type and a young patient which makes MEC to be more challenging to diagnose and treat. Minimally invasive surgical techniques, such as Uniportal video-assisted thoracic surgery (VATS) sleeve lobectomy, have gained popularity recently because of their advantages over open surgery, which include lower morbidity and faster recovery. We have used Uniportal video-assisted thoracic surgery as minimally invasive approach that can also reduce recovery time and scarring, which may provide insights into treatment options. Uniportal VATS through its single small incision, minimizes chest wall trauma, resulting in markedly less postoperative pain, reduced need for opioids, faster return to normal activities, and improved cosmetic outcomes [16,17]. U-VATS bronchoplasty delivers safe, parenchyma-sparing resection and reconstruction for intrabronchial MEC with preserved lung function and excellent outcomes [18]. Our patient had pulmonary MEC with locally invasive intrabronchial lesion as shown in her bronchoscopy and CT scan; therefore, we have performed U-VATS sleeve lobectomy with right main bronchoplasty to preserve her lung. The anastomosis was tension-free with continuous suturing to minimize complications and promote healing. We also performed mediastinal lymph node dissection to ensure oncological radicality and optimal outcomes. Mis-diagnosis will change the way of the management. Thus mis-treatment of the patient will change the lifestyle of the patient both somatically and psychologically. The SCARE guidelines for 2025 are adhered to in this case study [12].

## 2 Case report

### 2.1 Patient information

**Fig. 1:**
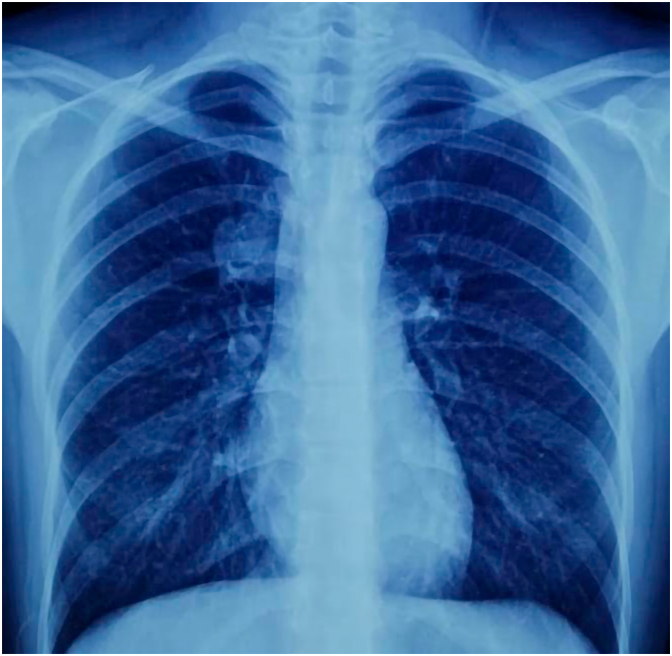
Pre- Operative CXR : Shows a round mass centrally located in the right upper zone.

A 25-year-old female presented with hemoptysis for 10 days. It was associated with mild cough and wheezing. The patient did not have shortness of breath or chest pain. The hemoptysis was not progressed with time and didn't cause upper airway obstruction. Her body mass index (BMI) was 18.5 kg/m^2^. She was a non-smoker. The patient had no chronic diseases or history of drug use. There were no significant features in the patient's family history. Upon arrival, the patient's temperature was 37.5 °C, blood pressure (BP) was 108/74 mmHg, respiratory rate was 19/min, pulse rate was 99 bpm, and her oxygen saturation (SpO₂) was 93 % on room air. She visited a pulmonologist for checking her condition. During physical examination by the pulmonologist: generally; she was alert, conscious, oriented, not dyspnic. But during chest examination; in inspection: there was no any chest wall deformity. On palpation; no any chest wall tenderness, mass or abnormalities. On percussion: lung sounds and resonance were normal. With Auscultation of her chest anteriorly and posteriorly, he found mild wheezing in the right upper zone and slightly decreasing of air entry in the right upper zone. He did not find any sings of recurrent chest infection. The Pulmonologist sent her for a Chest X-Ray (Fig. 1). He found a round mass in the right upper zone; therefore, he decided to do a Computerized Tomography (CT-Scan) of chest for more detail in the texture and the site of the mass. On January 16, 2025, she took a chest CT scan and it showed a 3.5 cm lesion in the right upper lobe locally invading the right main bronchus, and it was not metastasized to any other organs of the body (Video 1). Her laboratory test results were normal. Her pulmonologist performed a fiberoptic bronchoscopy (FOB); showed an intrabronchial lesion in her right main bronchus, looks like a vascularized mass and same as carcinoid tumor in its surface, which occluded right main bronchus and the fiberoptic bronchoscope can't pass it to see middle and lower bronchus well (Video 2), and a biopsy was taken and sent for histopathological examination with bronchial wash and brush, but the result was suspicious to be carcinoid tumor. Therefore, he decided to do endobronchial ultrasound (EBUS) (Video 3), and the result was inconclusive, also it was suspicious and suggested as monomorphic neuro-endocrine carcinoid tumor. But because the sample size of the biopsy of both Bronchoscopy and EBUS were small material; the Immunohistochemistry was inconclusive, therefore the pathologist asked for excision of the tumor. Finally, we decided to perform surgery for her.

### 2.2 Surgical intervention

On February 10, 2025, in the supine position, under general anesthesia, a double-lumen tube was inserted by assisting of fiberoptic bronchoscopy for confirmation of the *endo*-tracheal tube in the bronchus. The patient was positioned in the left lateral decubitus, which is the standard position. Her right elbow was positioned at a 90-degree angle. A tube or rolled sheet was placed below the axilla to widen the intercostal space. After disinfection of the chest from anterior axillary line till the tip of shoulders and posteriorly till lower part of left paravertebral area, the patient covered by sterilized towels. Through a 2–3 cm transverse incision in the 4th intercostal space at the mid-axillary line. We introduced a straight rigid thoracoscope with a 10 mm diameter and 30-degree angle as standard for adults. After the scope entered and adhesiolysis was performed, there was adhesion at the site of the biopsy that was taken by EBUS (Video 4), then we inoculated the mass in the right upper lobe, after good dissection we found a hard lesion locally invading the right main bronchus. We opened the right main bronchus to remove any remaining part of the tumor (Video 5). After removing the mass and obtaining a clear margin from the upper bronchus, the sample was sent for histopathology. The right main bronchus was sutured with polypropylene 3/0 (Video 6) ensuring a tension-free anastomosis with continuous sutures to minimize complications and promote healing. Then we dissected and separated the right upper lobe pulmonary artery branches and the upper pulmonary vein, we performed stapling of the pulmonary arteries and veins by vascular reloads, and the remaining pulmonary parenchymal tissue was stapled using advanced stapler with medium sized reloads, then the right upper bronchus was finally dissected and separated completely stapled by bronchial reloads for completing the sleeve upper lobectomy. We sent the lobe for histopathological examination. We performed Mediastinal lymph node dissection, we checked all the lymph nodes from paratracheal, subcarinal and around the right main bronchus, we retrieved about 11 nodes from the (stations 2R, 4R, 7, 10R, 11R). We have sent all the lymph-nodes for histopathology. We checked the site of the suturing for any air leak by using of normal saline and asking the anesthesiologist to inflate the lung, there was no any air leak and the lung was inflated well. After good hemostasis, we placed a single chest drain 28 French, we fixed the drain and sutured the incision in layers. Good dressing performed.

### 2.3 Diagnostic testing

Mucoepidermoid carcinoma (MEC) is a mainly salivary gland tumor that rarely occurs in the other areas, like lungs. MEC is usually made of 3 types of cells which are mucin-secreting cells, squamate cells and intermediate cells. It may be diagnosed by histopathology, immunohistochemistry and genetic study. Our case was a MEC in the lung of a young lady, it was locally invaded intra-bronchially and it was a single cell type not 3 cell type which gives the features of adenocarcinoma. Our patient was young and she had subacute hemoptysis, she was not smoker; therefore, review of the slides was mandatory. Also, it may have differentiation with carcinoid tumor but all the neuroendocrine markers were negative for the tumor. The diagnosis of such cases is significant because the style of the management will change, the outcome will change and the prognosis is totally different. FISH analysis had great role in diagnosing of MEC in our patient because the immunohistochemistry was not typical for adenocarcinoma and MEC. MEC of the lung by surgical excision has excellent prognosis but adenocarcinoma needs more workup and staging and the prognosis is quietly different. (See Timeline Table.)

**Timeline Table:** Timeline of the proceedings.

Proceedings	Detections	Interventions
In 12th January 2025.	Hemoptysis	
First CXR in 15th January 2025.	There was a round mass in the right upper zone centrally located.	
First CT in 16th January 2025.	Showed a mass in the right upper lobe and extended to the right main bronchus.	
First Fiberoptic Bronchoscopy in 28th January 2025.	Showed a mass in the right main bronchus and extending to the right upper lobe.	Biopsy was taken and sent for histopathological examination. The result was suspecting carcinoid tumor.
EBUS was performed in 4th February 2025.		Biopsy was taken and sent for HPE. The result was not conclusive only suspecting **neuro- endocrine carcinoid tumor.**
U-VATS in 10th February 2025.	The lesion was in the pulmonary parenchyma of the right upper lobe and locally invaded intra-bronchial in the right upper bronchus.	Right Sleeve Upper lobectomy was performed.
Regarding the HPE:- 1- The HPE after lobectomy in Mihrabani Surgical Hospital was in 17th February 2025. 2- The reviewed biopsy in Dublin was in 3rd March 2025.	The result was like low grade monomorphic adenocarcinoma. The slides were reviewed in an advanced laboratory Center. FISH analysis using the Zytolight probe revealed a positive result for MAML2 gene rearrangement, the diagnosis was mucoepidermoid carcinoma.	
Regarding the follow up: Forty days post-operatively (In 23rd March 2025) Seventy-five days post-operatively (In 28th April 2025)	The oncologist sent her for a PET -CT scan, it showed no more lesion in the chest. The entire body was clear from the disease.	
	There was two times hemoptysis (fresh blood) within 3 days.	We performed a new Fiberoptic Bronchoscopy for her, the tracheobronchial tree was erythematous, no any lesion, leak or recurrence. Only a small ulcer was found in the secondary carina of the left lower lobe. She received treatment, and now she is doing well. Bronchoalveolar lavage was taken and sent for cytology, culture of fungi, Acid Fast Bacilli (AFB) and Genetic study of Cytomegalovirus; all were normal.

#### 2.3.1 Histopathological findings

##### 2.3.1.1 Macroscopic (gross) findings

The specimen was right upper lobe of a lung measured 14x6x5 centimeters. The cut section mass was a white gray mass; measured 4.5 × 1.5 × 1 centimeters, multiple pieces was taken from the mass with 2 bronchial excision margin: 1 cm and 1.5 cm respectively, and rest of the lung.

##### 2.3.1.2 Microscopic features

**Fig. 2:**
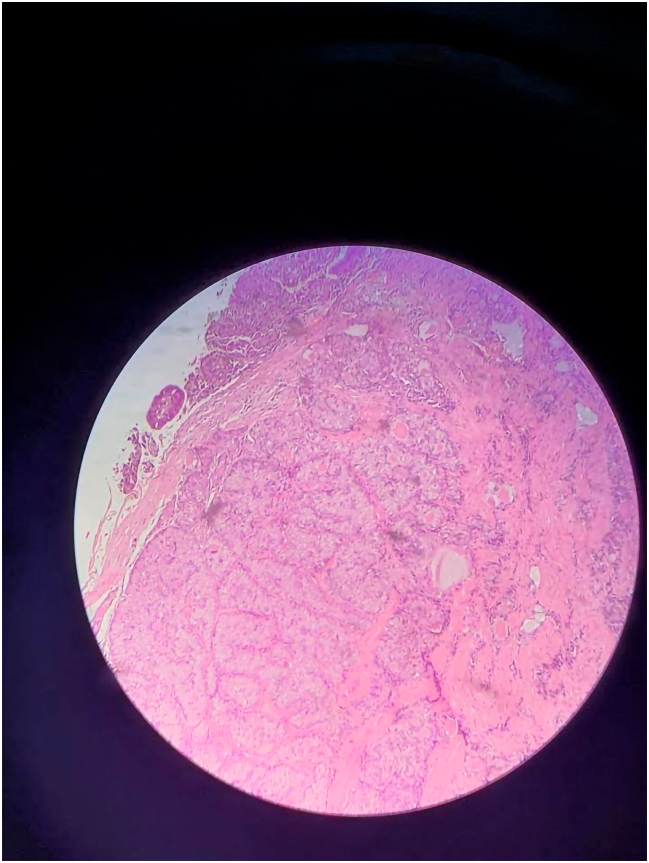
Shows round nuclei, few with prominent nucleoli and eosinophilic cytoplasm. Scattered cells showed intracytoplasmic mucin.

The tumor sections revealed endobronchial polypoid growth, with tumor infiltration into the surrounding parenchyma. The tumor was characterized by large, smooth, rounded solid cellular nests separated by thin fibrovascular septa. Focal lymphocytic infiltration was noted, but desmoplastic stroma was absent. Peripheral palisading and trabecular patterns were observed. The tumor cell cytoplasm was predominantly eosinophilic, with areas of clear cells. Mucin-filled spaces were highlighted by PAS and DPAS stains. The tumor cell cytology was uniform, with no pleomorphism (Fig. 2).

All the neuroendocrine markers were negative for the tumor. Immunohistochemical staining showed diffuse positivity for AE1/3 and CK7, while SOX-10 and DOG-1 were negative. P63 was negative.

##### 2.3.1.3 Fluorescent In-Situ Hybridization (FISH) Analysis

FISH analysis using the Zytolight probe revealed a positive result for MAML2 gene rearrangement, supporting the diagnosis of mucoepidermoid carcinoma. The analysis was performed, and 74 % of the cells showed break-apart signals, indicating rearrangement involving the MAML2 locus (11q21).

### 2.4 Follow-up

**Fig. 3:**
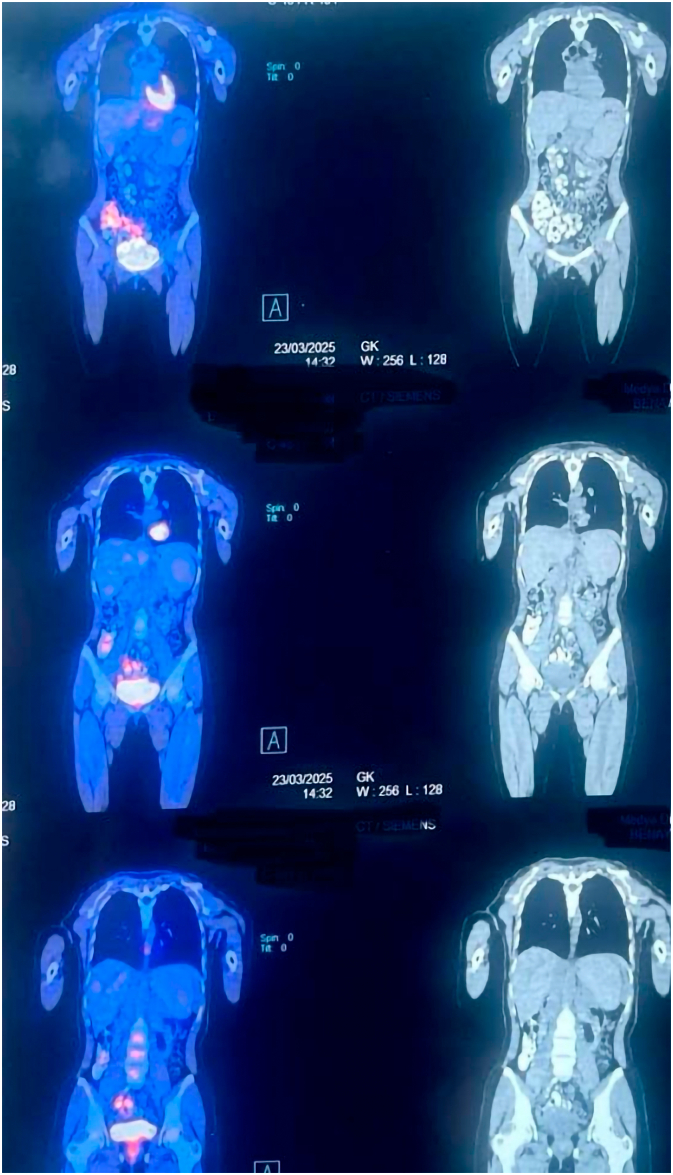
Post-Operative PET- CT Scan of the chest shows no more lesion in the right upper lobe and the right main bronchus.

**Fig. 4:**
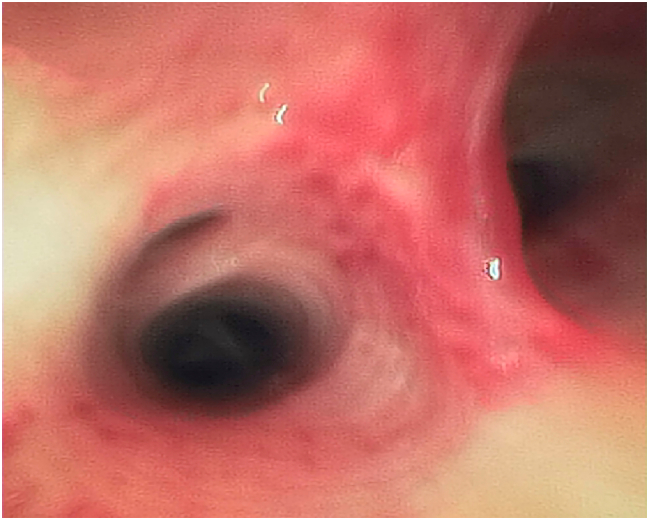
Fiber Optic Bronchoscopy shows an ulcer in the secondary carina of the left lower bronchus.

**Fig. 5:**
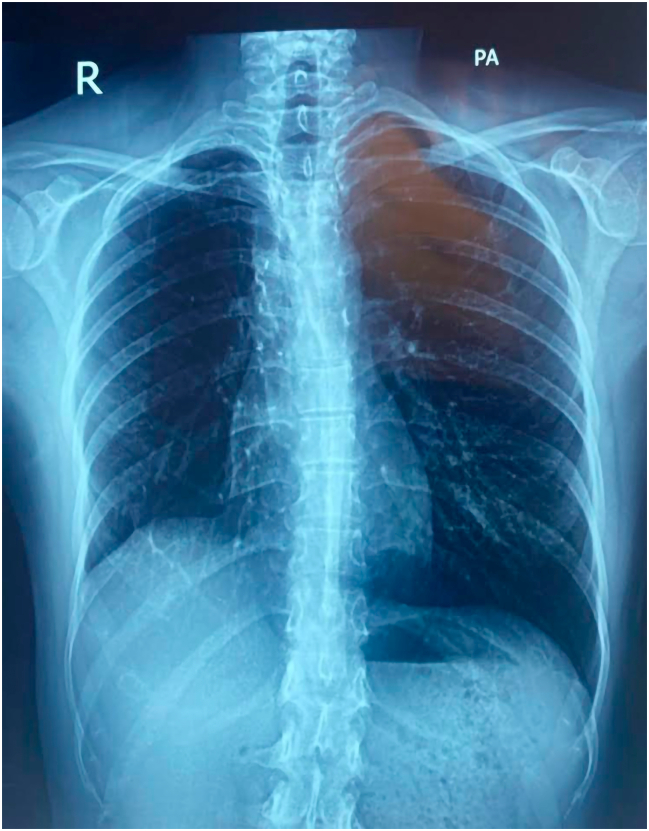
Post- Operative CXR; appears normal, no more lesion in the right upper lobe.

The patient was referred to the oncologist for any adjuvant chemotherapy or further investigations. On 23 March 2025; the oncology team sent the patient for a positron emission tomography (PET)-CT scan for any remaining lesions or metastasis (Fig. 3). The PET scan result was clean, and there were no any other lesions (Timeline table). Because of total resection (R0) and no mediastinal lymph node metastasis, the oncologists decided not to give her chemotherapy at this time. Two and half months after the surgery, the patient presented with recent hemoptysis 2 times within 3 days (Timeline Table). We performed fiberoptic bronchoscopy and there was only erythematous trachea - bronchial tree, and we found a small ulcer in the secondary carina of the left lower lobe (Fig. 4), while the site of the operation was totally free from ulcer, leak and any recurrence of the disease. And we sent her for a new CXR (Fig. 5), it was normal, the lungs were fully expanded and free from any lesion, hemothorax, pneumothorax, no any pleural effusion or atelectasis.

## 3 Discussion

Since MEC frequently manifests with vague symptoms, diagnosing it can be challenging [1]. Based on their histological appearance, mucoepidermoid tumors are categorized as either high-grade or low-grade [4,5,10]. In our instance, the patient's subacute onset of hemoptysis with mild cough and wheezing led to additional research. Her CT-Scan shows a right upper lobe lesion that was locally invaded intra-bronchial lumen. The fiberoptic bronchoscopy showed an intrabronchial lesion and the biopsy was taken and sent for the histopathological examination, even EBUS was performed for the patient but the results was suspicious and it was near to be carcinoid tumor, but after the upper lobectomy MEC was found by histopathological analysis. The slides were sent to another advanced center in another country, for sophisticated laboratory analysis, and genetic testing was used to confirm the findings. The goal of this step was to avoid the possible loss of pulmonary function or deformities of the chest wall that could arise from large lung resections. More selective surgeries are made possible by early diagnosis [6]. The most common procedure for MEC is lobectomy, but surgical resection is still the mainstay of the treatment [2,4]. However, there is a considerable risk of morbidity and mortality with traditional open thoracotomy. For the treatment of lung cancer, minimally invasive surgical methods like Uniportal VATS have grown in popularity recently [11]. Compared to open surgery, uniportal VATS sleeve lobectomy is a novel procedure that shortens recovery times and minimizes complications [2,3]. With outstanding long-term results, tracheobronchial sleeve resection is regarded as the best surgical option for intrabronchial tumors [13]. Uniportal VATS bronchoplasty is safe, will save the lung from more resection by parenchyma-sparing and reconstruct the bronchus like intrabronchial MEC with preserving lung function and excellent outcomes (18). Less invasive procedures like Uniportal Video-Assisted Thoracoscopic Surgery (UVATS) are increasingly utilized for mucoepidermoid carcinoma due to significant clinical benefits, including reduced postoperative pain, faster recovery times, and shorter hospital stays, compared to traditional open surgery or multiport VATS [19,20]. With sleeve lobectomy and right main bronchus reconstruction, we have saved the right lung from pneumonectomy and we preserved the lung function with great results.

In this rare case we have performed sleeve right upper lobectomy via Uniportal VATS. The systematic lymphadenectomy was performed (stations 2R, 4R, 7, 10R, 11R). Zhu et al. reported a median of 13 nodes (range 3–18) dissected via uniportal VATS [21]; we retrieved 11 nodes, all were negative, consistent with the low-grade biology of MEC and ensuring oncological radicality.

The histopathology of MEC appears as 3 cell types; epidermoid cells, intermediate cells and mucocytes [14,15]. But the histology of our case was like 1 cell type; It was not typical for each adenocarcinoma and MEC. Even the immunohistochemistry was not typical for adenocarcinoma and MEC. P63 was also negative. Two subspeciality pathologists confirmed the diagnosis via genetic study with FISH; which showed gene rearrangement involving MAML2 locus (11q21). The pathologists should not depend on one way of diagnosis, and even should not depend on the similarity in the texture of the tissues. Because the patient undergoes mis-treatment and her lifestyle will be definitely changed both somatically and psychologically.

## 4 Conclusion

In conclusion, this case demonstrates how a young woman's intrabronchial mucoepidermoid carcinoma was successfully treated with a Uniportal VATS sleeve lobectomy. The pathologists must be aware of diagnosis of such rare cases, because such young lady may be mistreated and her life style will be changed totally both somatically and psychologically. The importance of early diagnosis, team-based care, and the potential of minimally invasive surgery for treating rare lung cancers are all highlighted by this unusual and complicated case.

The following are the supplementary data related to this article.

Video 1 Pre-Operative CT Scan of Chest; shows a round big mass in the right upper lobe invafing right main bronchus. Video 1

Video 2 Pre-Operative Bronchoscopy; shows a big mass in the right main bronchus, does not allowing the scope to pass under it. Video 2

Video 3 Pre-Operative EBUS; show as round big mass in the right upper lobe invading right main bromchus. Video 3

Video 4 U-VATS; shows adhesion of the right main bronchus with right upper lobe and azygus vein at the site of the EBUS. Video 4

Video 5 U-VATS; shows resection of the invaded part of the mass from the right main bronchus. Video 5

Video 6 U-VATS; shows suturing of the right main bronchus after lobectomy. Video 6

## Patient consent

Written informed consent was obtained from the patients for publication of this case repot and accompanying images. A copy of the written consent is available for review by the Editor-in-Chief of this journal on request.

## Ethical approval

This case report is exempt from ethical approval committee of our university (University of Kurdistan Hawler- school of Medicine). The name of the committee is Biomedical Research Center and the Ethical Code is [UKH-AF-001].

The reason; because it doesn't involve sensitive information.

## Guarantor

Rawand Abdulrahman Essa (Rawandessa86@gmail.com)

## Research registration number

Not applicable.

## Provenance and peer review

Not commissioned, externally peer-reviewed.

## Funding

This research did not receive any specific grant from funding agencies in the public, commercial, or not-for-profit sectors.

## Author contribution

RAE and DSS conceived and designed the study. RAE, DSS, KSH, SSA, RHS and LRS collected, analyzed, and interpreted the data. RAE, DSS, KSH, SSA, RHS and LRS drafted the manuscript, which was critically revised and approved by all the authors.

## Conflict of interest statement

The authors declare no conflict of interest.

## Data Availability

All relevant data are within the manuscript.
